# Supplementary material for: Local spectroscopy of a gate-switchable moiré quantum anomalous Hall insulator
Source: Nat Commun. 2023 Jun 16;14:3595. doi: 10.1038/s41467-023-39110-3 (PMC10275868; doi:10.1038/s41467-023-39110-3)
Supplement: Supplementary file 1 — Supplementary Information [file 41467_2023_39110_MOESM1_ESM.pdf]

Supplementary Information: Local spectroscopy of a gate-switchable moiré quantum anomalous Hall insulator

Canxun Zhang, Tiancong Zhu, Tomohiro Soejima, Salman Kahn, Kenji Watanabe, Takashi Taniguchi, Alex Zettl, Feng Wang, Michael P. Zaletel, Michael F. Crommie

Table of Contents

Supplementary Note 1	Identifying the three stacking regions of tMBLG	1
Supplementary Note 2	Comparison between transport and STM/STS measurements	2
Supplementary Note 3	Response of the $\nu = 3$ correlation gap size to an applied out-of-plane magnetic field	3
Supplementary Note 4	Comparison between QAH phases in tMBLG and MA-tBLG	3
Supplementary Note 5	Determining the correlation gap position	4
Supplementary Note 6	Theoretical model and calculations	4
Supplementary Figure 1	Optical microscope image of the tMBLG device	9
Supplementary Figure 2	Parameter space in transport and STM/STS measurements	9
Supplementary Figure 3	Additional data for correlated insulating states at $\nu = 2, 3$	10
Supplementary Figure 4	Topological behaviour of the $\nu = 3$ state at $B = 0.2$ T	11
Supplementary Figure 5	Gate-dependent dI/dV without the $\nu = 3$ correlation gap	11
Supplementary Figure 6	Topological behaviour of the $\nu = 3$ state for the large-strain region	12
Supplementary Figure 7	Extracting correlation gap positions from gate-dependent dI/dV	12
Supplementary Figure 8	Strain tuning Hartree-Fock band structure for tMBLG	13
Supplementary Figure 9	Calculating bulk magnetization in the presence of electron-electron interaction	13

Supplementary Note 1: Identifying the three stacking regions of tMBLG

STM topographic images of tMBLG (Fig. 1b, Fig. 3e,g) typically show three distinct regions within each moiré unit cell, which we call "bright", "intermediate" and "dark" based on their

apparent heights for $|V_{\mathrm{Bias}}| > 100$ mV. We determined their local stacking orders by analysing structural as well as electronic contributions to the apparent height. AAB stacking in tMBLG is energetically unfavourable (due to strong repulsion between the carbon atoms in the "AA" layers) and exhibits an out-of-plane structural displacement, so we identify the "bright" region as AAB stacking. ABC and BAB stackings, on the other hand, have similar binding energies to each other and similar structural heights, so atomic structure alone is inadequate to explain the difference between the observed "intermediate" and "dark" regions. We have calculated the local density of states (LDOS) at the ABC and BAB sites using the continuum model (see Supplementary Note 6) and integrated them from –200 to 0 meV (simulating a negative V_{Bias}) or from 0 to 200 meV (simulating a positive V_{Bias}), and find that ABC always displays a higher intensity than BAB. Since a larger integrated LDOS corresponds to a higher apparent height in constant-current STM measurements, we identify the "dark" region as corresponding to BAB stacking and the "intermediate" region ABC stacking.

Supplementary Note 2: Comparison between transport and STM/STS measurements

In transport measurements on tMBLG the graphene layers are usually encapsulated between two pieces of hBN with a top gate and a bottom gate on two sides.[1-4] This allows independent tuning of carrier density n and out-of-plane electric field $\mathbf{E} = (0, 0, E)$ in the graphene stack through combination of top and bottom gate voltages. The electric field creates a potential difference $\Delta_U = eEd_{\mathrm{a}}$ between adjacent graphene layers ($d_{\mathrm{a}} = 0.33$ nm is the inter-layer distance) which impacts the shape and alignment of flat bands and hence the correlated states of tMBLG. Supplementary Figure 2 shows a schematic of R_{xx} as a function of both n and E in a typical transport measurement of tMBLG (from Ref. [1]). Correlated insulating states emerge at $\nu = 1, 2, 3$ over a finite range of E, demonstrating electric-field-tuning of correlation effects in tMBLG. In our STM/STS measurement geometry, a dedicated top gate is not achievable due to the existence of the STM tip. Both n and E are controlled by the back-gate voltage V_{G} and cannot be independently tuned (tip-induced gating is not observed in our experiment due to deliberate work function matching between graphene and the tip material). The different chemical environment of exposed carbon atoms in the top layer and those in contact with hBN in the bottom layer leads to an additional inter-layer potential difference Δ_{U0}. The overall inter-layer potential difference is $\Delta_U = \Delta_{U0} + eEd_{\mathrm{a}}$ where E is directly related to n by $E = \frac{ne}{2\varepsilon_{\mathrm{eff}}\varepsilon_0}$ ($\varepsilon_{\mathrm{eff}}$ is the effective out-of-plane dielectric constant of tMBLG). The parameter space in STM/STS measurements therefore corresponds to a "diagonal" line-cut in Supplementary Fig. 2,

which touches the $\nu = 2$ and the $\nu = 3$ correlated insulating states while missing the $\nu = 1$ one. This explains why correlation gaps appear in dI/dV at $\nu = 2$, 3 but not at $\nu = 1$ in our data.

Supplementary Note 3: Response of the $\nu = 3$ correlation gap size to an applied out-of-plane magnetic field

The size of the $\nu = 3$ correlation gap should be modified by application of an out-of-plane magnetic field $\mathbf{B} = (0, 0, B)$ due to the Zeeman energy associated with non-zero orbital magnetic moments, analogous to the case in Ref. [5]. We can estimate the magnitude of this effect by considering single-particle effects and assuming that each $C = +2$ sub-band in the CFB manifold has a magnetization of $m_{C\,=\,\pm 2}$ as defined in Supplementary Note 6. For the K+ valley-polarized $C_{\mathrm{tot}} = +2$ state (Fig. 4a), the doubly-occupied $C = +2$ sub-bands then shift downward by $B\left|m_{C\,=\,\pm 2}\right|$ (assuming $B > 0$), whereas both the occupied and the unoccupied $C = -2$ sub-bands shift upward by the same amount. The correlation gap should remain the same since it lies between the two $C = -2$ sub-bands. For the K– valley-polarized $C_{\mathrm{tot}} = -2$ state (Fig. 4b), on the other hand, the correlation gap (which lies between the doubly occupied $C = -2$ sub-bands and the unoccupied $C = +2$ sub-band) should decrease by $2B\left|m_{C\,=\,\pm 2}\right|$. We estimate this shift to be less than 2 meV at $B = 2$ T based on calculated $m_{C\,=\,\pm 2}$ values (Supplementary Fig. 9b-d), and so this effect is obscured by thermal and instrumental broadening and is not significant in our data.

Supplementary Note 4: Comparison between QAH phases in tMBLG and MA-tBLG

In addition to tMBLG, the QAH effect has also been observed in magic-angle twisted bilayer graphene (MA-tBLG) aligned with an hBN substrate.[6] The underlying physics of the QAH phases in tMBLG and hBN-aligned MA-tBLG are similar, but two major differences have been reported in transport experiments. First, the Hall conductance is quantized to $\pm e^2/h$ for MA-tBLG and $\pm 2e^2/h$ for tMBLG, since the Chern number for QAH states of MA-tBLG is ±1 while for tMBLG it is ±2. Second, gate-induced Chern number switching has only been observed in tMBLG. This likely depends on the details of band structure, as discussed in Ref. [7]. In terms of local spectroscopy, signatures of Chern insulating behaviour have been detected via STM/STS in hBN-aligned MA-tBLG under low magnetic fields,[8] but no systematic study on the QAH phase has been performed yet.

Supplementary Note 5: Determining the correlation gap position

We determined the V_G (ν) values at which the correlation gap appears (Fig. 3f,h) using the following procedure. Supplementary Figure 7a shows normalized dI/dV at V_{Bias} = 0 mV (E_F) as a function of V_G for different B values (this is for a small-strain region; the data is the same as in Fig. 2j). The single dip at ν = 3 for B = 0.0 T and two separate dips above and below ν = 3 for finite B signify the formation of correlation gaps at these filling factors. The dip features, however, are often accompanied by prominent peaks indicated by red arrows in Supplementary Fig. 7a. To understand their origin, we carefully examine Fig. 2d-i (Fig. 2d is reproduced with slightly different color scale in Supplementary Fig. 7b as an example) and focus on the features that give rise to the dip-adjacent peaks in Supplementary Fig. 7a. As V_G increases, they rapidly shift to higher energies (highlighted by the dashed box in Supplementary Fig. 7b), which is the opposite direction compared to the movement of CFB and VFB peaks. This suggests that they likely arise from tip-induced charging[9,10] and do not reflect the actual LDOS in tMBLG. For every curve in Supplementary Fig. 7a, we thus exclude the dip-adjacent peaks and perform linear fitting on the two sides of each dip (fitted lines are shown for B = 0.0 T as an example). The data points in Fig. 3f indicate intersection of fitted lines and the error bars come from the fitting errors. The same procedure is conducted for data from the large-strain region (Supplementary Fig. 7c; the data is the same as in Supplementary Fig. 6f) and the results are plotted in Fig. 3h.

Supplementary Note 6: Theoretical model and calculations

Continuum model of tMBLG. Our calculations were based on the Bistritzer–MacDonald continuum approach to moiré structures.[11,12] Here the monolayer graphene is modelled using a two-band tight-binding model with t_0 = 2.8 eV while the Bernal-stacked bilayer is modelled using a four-band model with t_0 = 2.61 eV, t_1 = 0.361 eV, t_3 = 0.283 eV, t_4 = 0.138 eV, Δ = 0.015 eV.[13] The bilayer is then rotated by angle θ and hybridized with the monolayer with intra-sublattice strength w_{AA} = 87.75 meV and inter-sublattice strength w_{AB} = 117 meV. The hetero-strain is taken into consideration as an artificial vector field, following the treatment in Ref. [14]. We further add a potential difference $\Delta_U = (12.6 + 4.69\,\nu)$ meV between adjacent graphene layers to account for the gate-induced out-of-plane electric field as well as the built-in asymmetry between top and bottom layers due to the presence of the hBN substrate (Supplementary Note 2). The resulting continuum model is truncated by keeping all states within a radius of 6 mini-Brillouin zones (mBZs) of the γ-point.

Decomposing orbital magnetization into bulk and edge components. When the chemical potential μ resides inside the $\nu = 3$ correlation gap, the total orbital magnetization can be decomposed into the bulk part which is independent of μ and the edge part which depends linearly on μ. There is no unique way of defining these two pieces. We find it convenient to use the following convention:

$$M_{\mathrm{bulk}} = \frac{e}{2\hbar}\sum_n \int d^2\mathbf{k}\,\epsilon^{ab}\,\mathrm{Im}\langle \partial_a u_{n,\mathbf{k}} | H_{\mathbf{k}} + \mathcal{E}_{n,\mathbf{k}} - 2\mathcal{E}_{\mathrm{max}}^{\mathrm{OC}} | \partial_b u_{n,\mathbf{k}} \rangle \quad (1)$$

$$M_{\mathrm{edge}}(\mu) = -\frac{e}{\hbar}\sum_n \int d^2\mathbf{k}\,\epsilon^{ab}\,\mathrm{Im}\langle \partial_a u_{n,\mathbf{k}} | \mu - \mathcal{E}_{\mathrm{max}}^{\mathrm{OC}} | \partial_b u_{n,\mathbf{k}} \rangle = \frac{e}{h} C_{\mathrm{tot}}(\mu - \mathcal{E}_{\mathrm{max}}^{\mathrm{OC}}) \quad (2)$$

where ϵ^{ab} is the antisymmetric tensor, $\mathcal{E}_{n,\mathrm{k}}$ is the single particle energy, and $\mathcal{E}_{\mathrm{max}}^{\mathrm{OC}}$ is the energy of the top of the occupied bands (i.e., the bottom of the correlation gap). This convention has the advantage that $M_{\mathrm{edge}}(\mathcal{E}_{\mathrm{max}}^{\mathrm{OC}}) = 0$, as befitting the interpretation that M_{edge} is the contribution from chiral edge modes. Appropriate care needs to be taken to compute these quantities on a discrete grid, as described earlier in Refs. [15,16].

Calculating M_{edge}. For a given band structure, the sign of M_{edge} always follows that of C_{tot}, while its magnitude reaches the maximum when $\mu = \mathcal{E}_{\mathrm{min}}^{\mathrm{UO}}$ ($\mathcal{E}_{\mathrm{min}}^{\mathrm{UO}}$ is the energy of the bottom of the unoccupied bands, i.e., the top of the correlation gap):

$$M_{\mathrm{edge}}\left(\mu = \mathcal{E}_{\mathrm{min}}^{\mathrm{UO}}\right) = \frac{e}{h} C_{\mathrm{tot}}\left(\mathcal{E}_{\mathrm{min}}^{\mathrm{UO}} - \mathcal{E}_{\mathrm{max}}^{\mathrm{OC}}\right) = \frac{e}{h} C_{\mathrm{tot}}\mathcal{E}_{\mathrm{g}} \quad (3)$$

where $\mathcal{E}_{\mathrm{g}}$ is the size of the correlation gap. To obtain $\mathcal{E}_{\mathrm{g}}$ values as a function of hetero-strain, we performed Hartree-Fock calculation in a momentum-space approach analogous to earlier Hartree-Fock studies of twisted graphene systems. Our code is an extension of the tBLG code used in Ref. [17], the twisted double bilayer graphene (tDBLG) code used in Ref. [18], and the tMBLG code used in Ref. [19]. The Coulomb interaction (screened by the graphene, the hBN/SiO_2 substrate and the metallic STM tip) is assumed to take the single-plane-screened form $V(\mathbf{q}) = \frac{e^2}{2\epsilon_{\mathrm{eff}}\epsilon_0 q}[1 - \exp(-2qd_{\mathrm{S}})]$ ($\varepsilon_{\mathrm{eff}} = 12$ and $d_{\mathrm{S}} = 20$ nm were chosen on phenomenological grounds). The Coulomb matrix elements are evaluated in the basis of the continuum band structure and projected into the six bands nearest to the charge neutrality per valley and spin, for a total of 24 bands. We then consider a Slater-determinant ansatz $|u\rangle$ that is diagonal in the mBZ momentum $\mathbf{k}$. Discretizing the model on a 16x16 $\mathbf{k}$-grid, the u's are iteratively adjusted to minimize the energy $\langle u|H|u\rangle$, using the optimal damping algorithm in Ref. [20] to achieve Hartree-Fock self-consistency. Supplementary Figure 8a-c shows the resulting Hartree-Fock tMBLG band structures for selected hetero-strain values. Here positive (negative) strain is defined as stretching (compressing) the bilayer and compressing (stretching) the monolayer (bilayer). Increasing the hetero-strain leads to a reduction in $\mathcal{E}_{\mathrm{g}}$ (Supplementary Fig. 8d) and also in

M_{edge} as plotted in Fig. 4d (positive strain is assumed in the main text, but the results remain qualitatively the same for negative strain).

Calculating M_{bulk}. As M_{bulk} is heavily dependent on the detailed band structure, a full calculation based on the Hartree-Fock approach is extremely costly and beyond our current capabilities. Instead, we adopt the phenomenological model proposed in Refs. [1,7] to take electron-electron interaction into consideration. For our purpose it is convenient to take an alternative decomposition of orbital magnetization

$$\begin{aligned} M_{\text{bulk}} &= \frac{e}{2\hbar}\sum_n \int d^2\mathbf{k}\,\epsilon^{ab}\,\text{Im}\langle \partial_a u_{n,\mathbf{k}} | H_{\mathbf{k}} + \mathcal{E}_{n,\mathbf{k}} - 2\mathcal{E}_{\text{max},n} | \partial_b u_{n,\mathbf{k}} \rangle \\ &\quad - \frac{e}{\hbar}\sum_n \int d^2\mathbf{k}\,\epsilon^{ab}\,\text{Im}\langle \partial_a u_{n,\mathbf{k}} | \mu - \mathcal{E}_{\text{max},n} | \partial_b u_{n,\mathbf{k}} \rangle \\ &= \sum_n m_n + \frac{e}{h}\sum_n C_n(\mu - \mathcal{E}_{\text{max},n}) \end{aligned} \tag{4}$$

where $\mathcal{E}_{\text{max},n}$ is the energy of the top of the n-th band and m_n can be interpreted as the total orbital magnetization of that band when the chemical potential is at $\mathcal{E}_{\text{max},n}$. We start from the single-particle band structure (for which the m_n's can be straightforwardly calculated using the dual state method of Ref. [16]) and analyse how electron-electron interaction impacts M_{bulk}. The primary effect of interaction is to introduce exchange energy difference between different sub-bands which causes spontaneous polarization in the spin-valley space. We expect the nature of each sub-band to remain unchanged to a good approximation. Therefore, we make several simplifying assumptions similar to those in Ref. [1] with slight modifications:

1. Interaction opens up a gap between the three occupied CFB– sub-bands and the one unoccupied CFB+ sub-band;
2. Interaction introduces an exchange-driven energy difference δ_{VFB} among the VFB sub-bands (which have valley-dependent $C = \mp 1$) (Supplementary Fig. 9a);
3. The energies of other bands remain unchanged;
4. Each m_n, evaluated at the top of each band after taking δ_{VFB} into account, remains unchanged due to interaction.

We can evaluate the total orbital magnetization when $\mu = \mathcal{E}_{\text{max}}^{\text{oc}}$ with these assumptions. Focusing on the K+ valley-polarized $C_{\text{tot}} = +2$ state, we get

$$
\begin{aligned}
M_{\text{bulk}} &= \sum_n m_n + \frac{e}{h}\sum_n C_n\left(\mathcal{E}_{\max}^{\text{OC}} - \mathcal{E}_{\max,n}\right) \\
&= m_{C\,=\,+2} + m_{C\,=\,+2} + m_{C\,=\,-2} \\
&+ \frac{e}{h}\left[(-1)\left(\mathcal{E}_{\max}^{\text{OC}} - \mathcal{E}_{\max,\text{VFB}}\right) + (-1)\left(\mathcal{E}_{\max}^{\text{OC}} - \mathcal{E}_{\max,\text{VFB}}\right)\right. \\
&+ \left.(1)\left(\mathcal{E}_{\max}^{\text{OC}} - \mathcal{E}_{\max,\text{VFB}}\right) + (1)\left(\mathcal{E}_{\max}^{\text{OC}} - \mathcal{E}_{\max,\text{VFB}} - \delta_{\text{VFB}}\right)\right] \\
&= m_{C\,=\,+2} - \frac{e}{h}\delta_{\text{VFB}}
\end{aligned}
\qquad (5)
$$

M_{bulk} for the $C_{\text{tot}} = -2$ state is the exact opposite. Here the first term (arising from single-particle effects) is parallel to M_{edge} while the second term characterizing electron-electron interaction strength is antiparallel to M_{edge}.

We find that $m_{C\,=\,+2}$ becomes larger as the hetero-strain is increased due to a redistribution of Berry curvature and band dispersion throughout the mBZ. δ_{VFB} is treated as a fitting parameter since it is difficult to accurately estimate the exchange coupling. Supplementary Figure 9b-d shows $M_{\text{bulk}}(C_{\text{tot}} = +2)$ as a function of the hetero-strain for different choices of δ_{VFB} when the twist angle θ is fixed at 1.26°, 1.28°, and 1.32°, respectively. In the main text we use $\delta_{\text{VFB}} = 13$ meV for $\theta = 1.26°$ (Fig. 4d), which best matches our observation that increasing hetero-strain causes $M_{\text{bulk}}(C_{\text{tot}} = +2)$ to flip from negative to positive and the system to change from switchable to non-switchable. For 1.30° $< \theta <$ 1.34°, only non-switchable QAH states have been observed experimentally (Fig. 3a), but this does not preclude a similar strain-induced transition since M_{bulk} could flip its sign for strains below 0.12%, the lowest amount observed within this angle range.

Supplementary References

1 Polshyn, H. *et al.* Electrical switching of magnetic order in an orbital Chern insulator. *Nature* **588**, 66-70 (2020). https://doi.org:10.1038/s41586-020-2963-8

2 Chen, S. *et al.* Electrically tunable correlated and topological states in twisted monolayer–bilayer graphene. *Nature Physics* **17**, 374-380 (2021). https://doi.org:10.1038/s41567-020-01062-6

3 Xu, S. *et al.* Tunable van Hove singularities and correlated states in twisted monolayer–bilayer graphene. *Nature Physics* **17**, 619-626 (2021). https://doi.org:10.1038/s41567-021-01172-9

4 He, M. *et al.* Competing correlated states and abundant orbital magnetism in twisted monolayer-bilayer graphene. *Nature Communications* **12**, 4727 (2021). https://doi.org:10.1038/s41467-021-25044-1

5 Li, S.-Y. *et al.* Experimental evidence for orbital magnetic moments generated by moiré-scale current loops in twisted bilayer graphene. *Physical Review B* **102**, 121406 (2020). https://doi.org:10.1103/PhysRevB.102.121406

6 Serlin, M. *et al.* Intrinsic quantized anomalous Hall effect in a moiré heterostructure. *Science* **367**, 900-903 (2020). https://doi.org:10.1126/science.aay5533

7 Zhu, J., Su, J.-J. & MacDonald, A. H. Voltage-Controlled Magnetic Reversal in Orbital Chern Insulators. *Physical Review Letters* **125**, 227702 (2020). https://doi.org:10.1103/PhysRevLett.125.227702

8 Oh, M. *et al.* Evidence for unconventional superconductivity in twisted bilayer graphene. *Nature* **600**, 240-245 (2021). https://doi.org:10.1038/s41586-021-04121-x

9 Zhao, Y. *et al.* Creating and probing electron whispering-gallery modes in graphene. *Science* **348**, 672-675 (2015). https://doi.org:10.1126/science.aaa7469

10 Velasco, J., Jr. *et al.* Visualization and Control of Single-Electron Charging in Bilayer Graphene Quantum Dots. *Nano Letters* **18**, 5104-5110 (2018). https://doi.org:10.1021/acs.nanolett.8b01972

11 Park, Y., Chittari, B. L. & Jung, J. Gate-tunable topological flat bands in twisted monolayer-bilayer graphene. *Physical Review B* **102**, 035411 (2020). https://doi.org:10.1103/PhysRevB.102.035411

12 Rademaker, L., Protopopov, I. V. & Abanin, D. A. Topological flat bands and correlated states in twisted monolayer-bilayer graphene. *Physical Review Research* **2**, 033150 (2020). https://doi.org:10.1103/PhysRevResearch.2.033150

13 Jung, J. & MacDonald, A. H. Accurate tight-binding models for the π bands of bilayer graphene. *Physical Review B* **89**, 035405 (2014). https://doi.org:10.1103/PhysRevB.89.035405

14 Bi, Z., Yuan, N. F. Q. & Fu, L. Designing flat bands by strain. *Physical Review B* **100**, 035448 (2019). https://doi.org:10.1103/PhysRevB.100.035448

15 Souza, I., Íñiguez, J. & Vanderbilt, D. Dynamics of Berry-phase polarization in time-dependent electric fields. *Physical Review B* **69**, 085106 (2004). https://doi.org:10.1103/PhysRevB.69.085106

16 Ceresoli, D., Thonhauser, T., Vanderbilt, D. & Resta, R. Orbital magnetization in crystalline solids: Multi-band insulators, Chern insulators, and metals. *Physical Review B* **74**, 024408 (2006). https://doi.org:10.1103/PhysRevB.74.024408

17 Bultinck, N. *et al.* Ground State and Hidden Symmetry of Magic-Angle Graphene at Even Integer Filling. *Physical Review X* **10**, 031034 (2020). https://doi.org:10.1103/PhysRevX.10.031034

18 Zhang, C. *et al.* Visualizing delocalized correlated electronic states in twisted double bilayer graphene. *Nature Communications* **12**, 2516 (2021). https://doi.org:10.1038/s41467-021-22711-1

19 Polshyn, H. *et al.* Topological charge density waves at half-integer filling of a moiré superlattice. *Nature Physics* **18**, 42-47 (2022). https://doi.org:10.1038/s41567-021-01418-6

20 Cancès, E. & Le Bris, C. Can we outperform the DIIS approach for electronic structure calculations? *International Journal of Quantum Chemistry* **79**, 82-90 (2000). https://doi.org:https://doi.org/10.1002/1097-461X(2000)79:2<82::AID-QUA3>3.0.CO;2-I

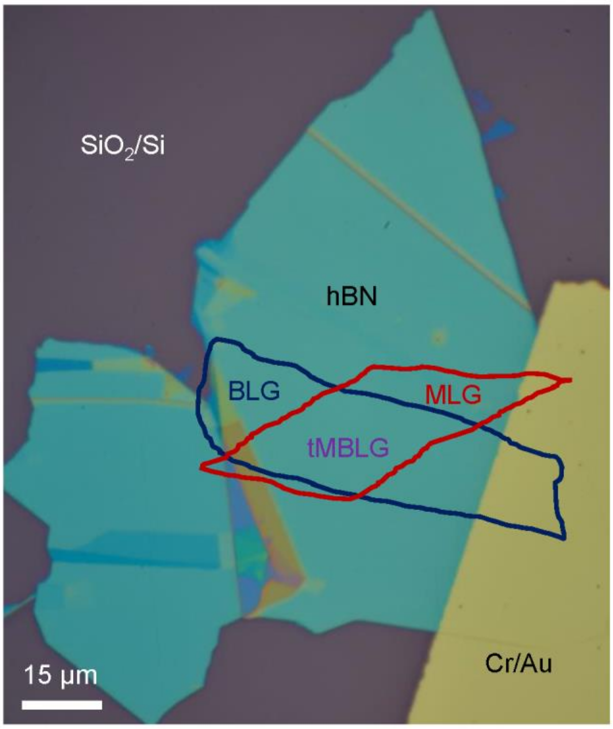

Supplementary Figure 1: Optical microscope image of the tMBLG device. MLG = monolayer graphene; BLG = Bernal-stacked bilayer graphene.

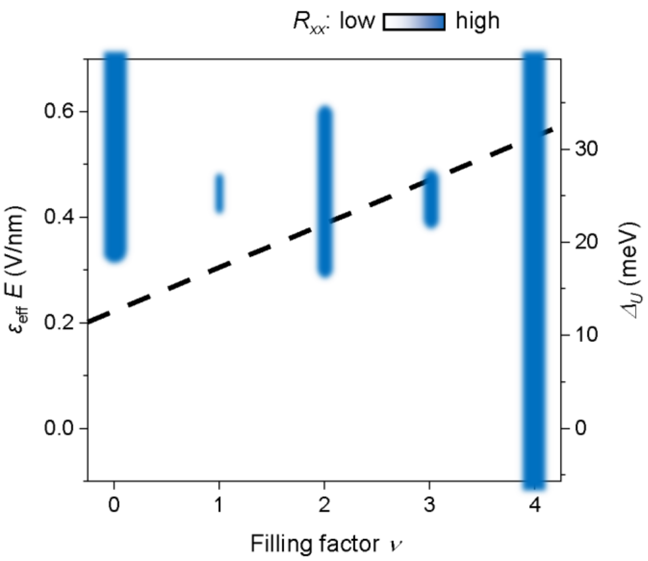

Supplementary Figure 2: Parameter space in transport and STM/STS measurements. The dark blue regions indicate insulating phases in transport measurement (adapted from Ref. [1]). The dashed line is the STM/STS parameter space. $\varepsilon_{\mathrm{eff}}$ is the effective dielectric constant of tMBLG, E is the out-of-plane electric field, and Δ_U is the corresponding inter-layer potential difference.

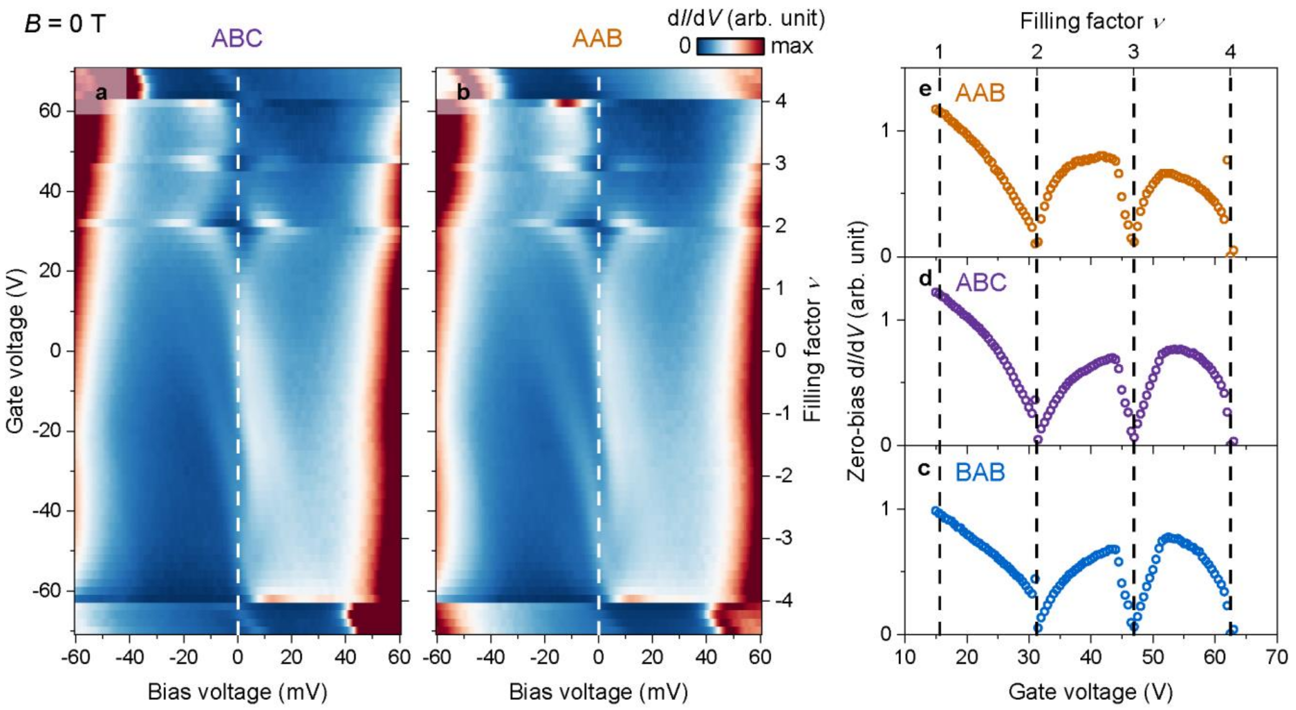

Supplementary Figure 3: Additional data for correlated insulating states at *ν* = 2, 3. a, Gate-dependent d*I*/d*V* density plot for the ABC stacking region over the gate range –70 V ≤ V_G ≤ 70 V. The vertical dashed line denotes the Fermi energy E_F. Spectroscopy parameters: modulation voltage V_{RMS} = 1 mV; setpoint V_{Bias} = 100 mV, I_0 = 1.55 nA for –70 V ≤ V_G ≤ –2 V; setpoint V_{Bias} = –100 mV, I_0 = 0.8 nA for 0 V ≤ V_G ≤ 70 V. **b**, Same as **a**, but for the AAB stacking region. Spectroscopy parameters: modulation voltage V_{RMS} = 1 mV; setpoint V_{Bias} = 100 mV, I_0 = 1.4 nA for –70 V ≤ V_G ≤ –2 V; setpoint V_{Bias} = –100 mV, I_0 = 0.8 nA for 0 V ≤ V_G ≤ 70 V. Splitting of the CFB peak around *ν* = 2, 3 is observed in spectra measured in all three regions (see also Fig. 1c). **c-e**, Normalized d*I*/d*V* at V_{Bias} = 0 mV (E_F) as a function of V_G (*ν*) for all three stacking regions. This type of plot is analogous to a transport conductance measurement since in both cases only electronic states near E_F are being probed. Dips going down to nearly vanishing d*I*/d*V* that are centred at *ν* = 2, 3, and 4 indicate emergence of insulating phases at these filling factors.

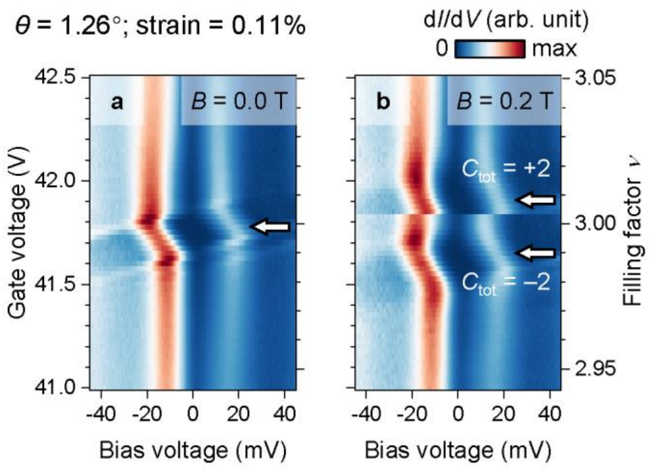

Supplementary Figure 4: Topological behaviour of the ν = 3 state at B = 0.2 T. Gate-dependent dI/dV density plot near ν = 3 at (**a**) B = 0.0 T and (**b**) B = 0.2 T (V_{RMS} = 1 mV; setpoint V_{Bias} = –60 mV, I_0 = 0.5 nA). Arrows indicate correlation gaps.

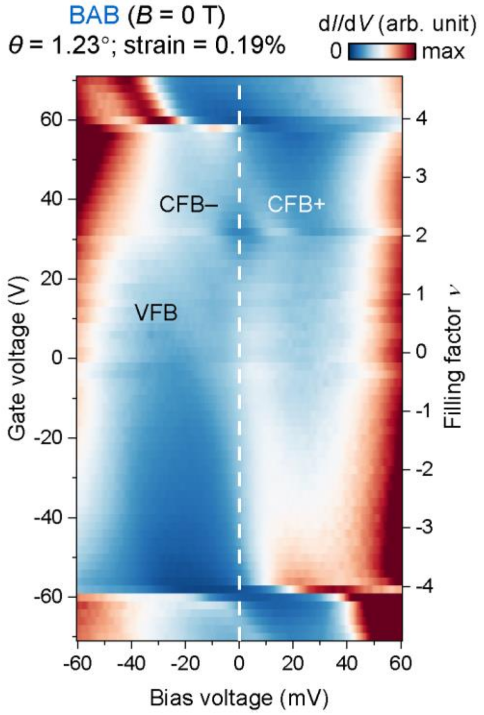

Supplementary Figure 5: Gate-dependent dI/dV without the ν = 3 correlation gap. These data were obtained in a region with θ = 1.23° and hetero-strain = 0.19%. The vertical dashed line denotes the Fermi energy. Spectroscopy parameters: modulation voltage V_{RMS} = 1 mV; setpoint V_{Bias} = 100 mV, I_0 = 2 nA for –70 V ≤ V_G ≤ –2 V; setpoint V_{Bias} = –100 mV, I_0 = 1 nA for 0 V ≤ V_G ≤ 70 V. VFB = valence flat band, CFB– = lower branch of conduction flat band, CFB+ = upper branch of conduction flat band.

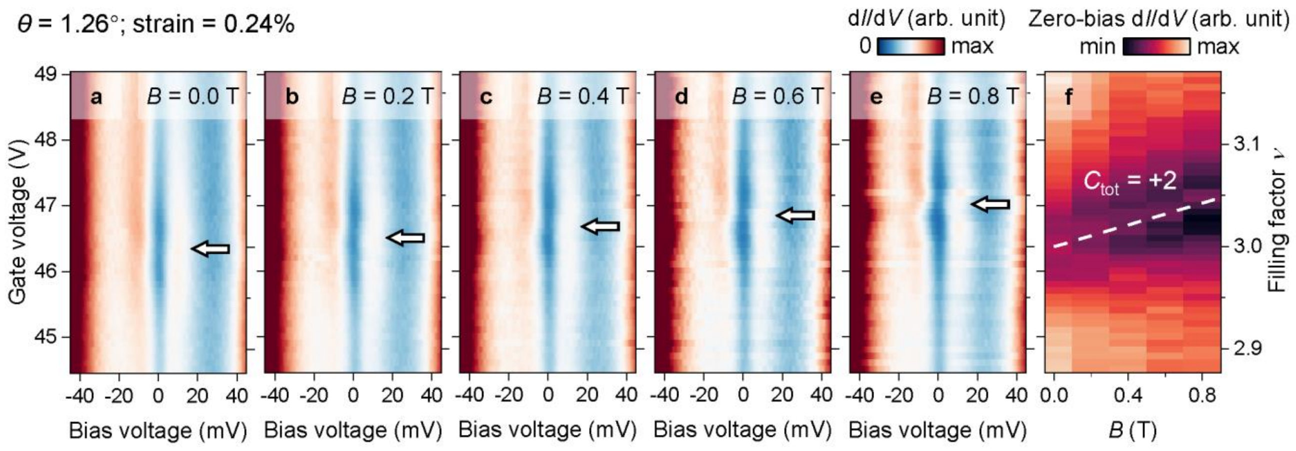

Supplementary Figure 6: Topological behaviour of the ν = 3 state for the large-strain region. a-e, Gate-dependent dI/dV density plot near ν = 3 for a region with θ = 1.26° and hetero-strain = 0.24% at (**a**) B = 0.0 T, (**b**) B = 0.2 T, (**c**) B = 0.4 T, (**d**) B = 0.6 T, and (**e**) B = 0.8 T (modulation voltage V_{RMS} = 1 mV; setpoint V_{Bias} = –60 mV, I_0 = 0.3 nA). Arrows indicate correlation gaps. **f**, Normalized dI/dV at V_{Bias} = 0 mV (E_{F}) as a function of V_{G} (ν) and B. The dashed line is a guide to the eye following the Středa formula with C_{tot} = +2.

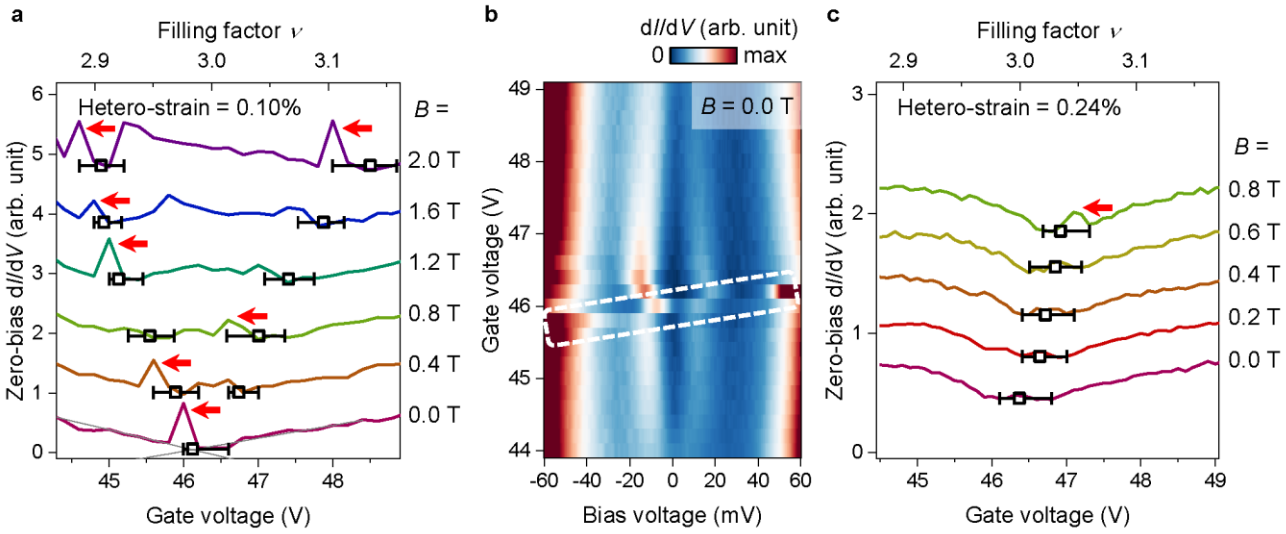

Supplementary Figure 7: Extracting correlation gap positions from gate-dependent d*I*/d*V*. a, Normalized dI/dV at V_{Bias} = 0 mV (E_{F}) as a function of V_{G} (ν) for different magnetic fields (small-strain region). Red arrows indicate tip-induced charging features. Squares with error bars are correlation gap positions. Fitted lines are plotted in grey for B = 0.0 T. **b**, Reproduction of gate-dependent dI/dV in Fig. 2d with the charging feature highlighted (dashed white box). **c**, same as **a**, but with data from the large-strain region.

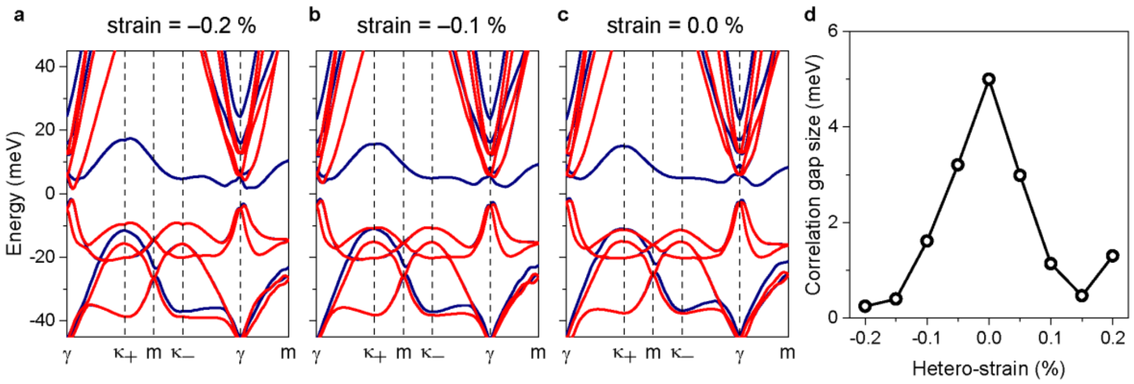

Supplementary Figure 8: Strain tuning Hartree-Fock band structure for tMBLG. a-c, Hartree-Fock band structures plotted along a high-symmetry line in the mBZ for hetero-strains of (**a**) –0.2%, (**b**) –0.1%, and (**c**) 0.0%. Red and blue curves represent sub-bands with different spins. **d**, Extracted correlation gap size as a function of hetero-strain.

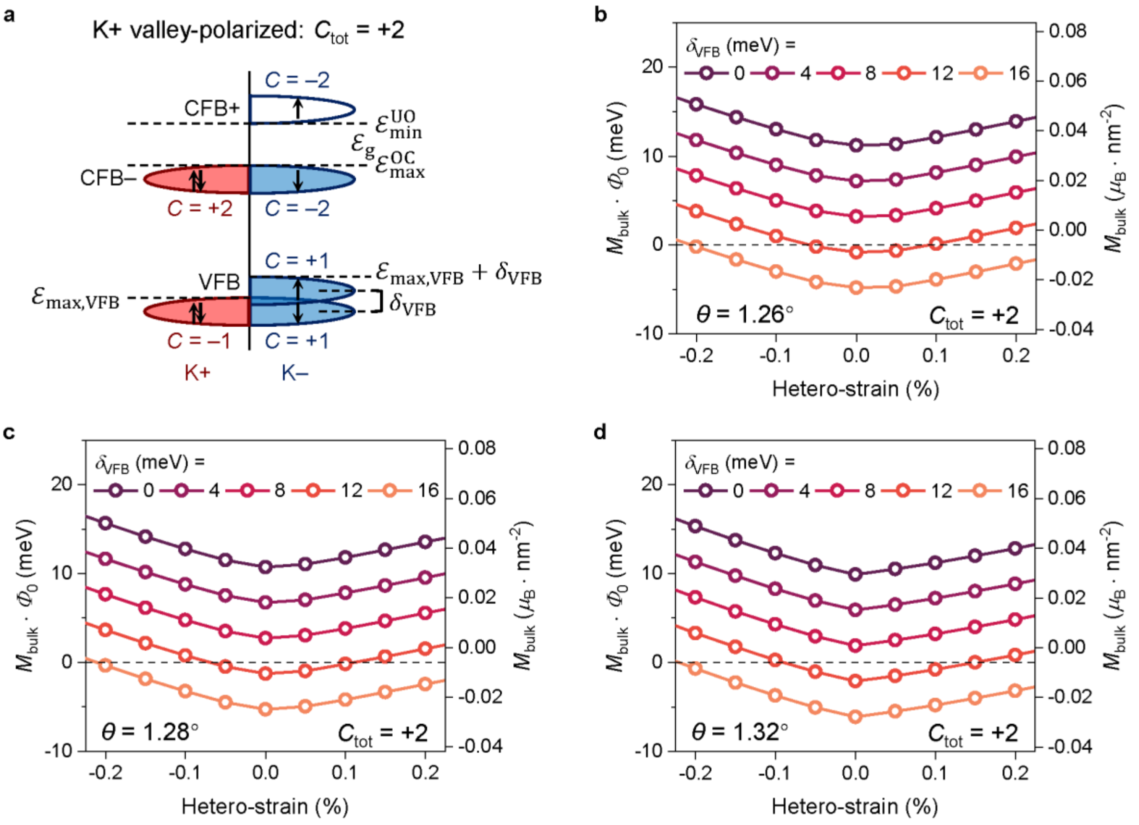

Supplementary Figure 9: Calculating bulk magnetization in the presence of electron-electron interaction. a, Energy configuration of the K+ valley-polarized state at $\nu = 3$ showing sub-bands from both the CFB manifold and the VFB manifold. δ_{VFB} is the interaction-induced energy offset among the VFB sub-bands. **b-d**, M_{bulk} for C_{tot} = +2 state as a function of hetero-strain for different choices of δ_{VFB} at (**b**) θ = 1.26°, (**c**) 1.28°, and (**d**) 1.32°.
